# Four new species of *Tremella* (Tremellales, Basidiomycota) based on morphology and DNA sequence data

**DOI:** 10.3897/mycokeys.47.29180

**Published:** 2019-02-27

**Authors:** Ying Zhao, Xin-zhan Liu, Feng-yan Bai

**Affiliations:** 1 State Key Laboratory for Mycology, Institute of Microbiology, Chinese Academy of Sciences, Beijing 100101, China 1 State Key Laboratory for Mycology, Institute of Microbiology, Chinese Academy of Sciences, Beijing 100101, PR China 2 Key Laboratory of Microbiology Diversity Research and Application of Hebei Province, College of Life Sciences, Hebei University, Baoding 071002, PR China Beijing China; 2 Key Laboratory of Microbiology Diversity Research and Application of Hebei Province, College of Life Sciences, Hebei University, Baoding 071002, China 1 State Key Laboratory for Mycology, Institute of Microbiology, Chinese Academy of Sciences, Beijing 100101, PR China Beijing China

**Keywords:** Basidiomycota, morphology, phylogeny, taxonomy, *
Tremella
*

## Abstract

In the present study, a total of 33 *Tremella* specimens in China were collected and examined using molecular phylogenetic analysis based on a combined sequence dataset of the nuc rDNA internal transcribed spacer (ITS) region and nuc 28S rDNA D1/D2 domain in conjunction with the morphological characters. Four new species, namely *Tremellabasidiomaticola*, *T.cheejenii*, *T.erythrina*, and *T.salmonea*, are newly described based on their distinct phylogenetic relationships and the comparison of morphological characters with known *Tremella* species. Our results indicate a high species diversity of *Tremella* waiting to be discovered.

## Introduction

*Tremella* Pers. has been traditionally considered to be the largest and most polyphyletic genus in Tremellaceae (Fell et al. 2000; Scorzetti et al. 2002; Sampaio et al. 2004; Boekhout et al. 2011; Millanes et al. 2011; Weiss et al. 2014; Liu et al. 2015a). The members of *Tremella* sensu lato are dimorphic fungi that contain both a haploid unicellular yeast stage and a dikaryotic filamentous stage. This genus is characterized by its mycoparasitic lifestyle and comprises species growing on the hymenium of Corticiales, Polyporales, Rhytismatales, and Russulales, on the mycelium of Russulales such as *Peniophora* and *Stereum*, in the basidiomata of Dacrymycetales, Polyporales, Russulales, and Trechisporales, on the perithecia of Diaporthales, Pleosporales, and Xylariales, as well as on lichens (Bandoni 1961; Reid 1970; Brough 1974; Zugmaier et al. 1994; Bandoni 1995; Roberts 1995, 1999, 2001, 2007; Roberts and deMeijer 1997; Diederich 1996; Torkelsen 1997; Chen 1998; Hauerslev 1999; Van Ryckegem et al. 2002; Pippola and Kotiranta 2008; Zamora 2009).

*Tremella* sensu lato includes approximately 90 species, more than half of which are known to exclusively parasitize specific lichenized fungal hosts (Diederich and Marson 1988; Diederich and Christiansen 1994; Diederich 1996, 2003, 2007, Sérusiaux et al. 2003; Kirk et al. 2008; Zamora 2009, Zamora et al. 2011, 2016; Millanes et al. 2012, 2014, 2015, 2016; Diederich et al. 2014; Kout et al. 2015; Lindgren et al. 2015; Westberg et al. 2015; Spirin et al. 2017). This genus splits into eight monophyletic groups in combination with several isolated species in Tremellales. Four clades have been emended, namely *Tremella* sensu stricto, *Carcinomyces*, *Naematelia*, and *Phaeotremella*, and one proposed as new genus, namely *Pseudotremella*. The other three clades consist of lichenicolous species that were defined as *Tremella* clade I, II, and III (Millanes et al. 2011; Liu et al. 2015a, b). Their taxonomy remains be determined until more robust phylogeny is resolved and further morphological characters are found. The basidiomata colour and shape of species belonging to *Tremella* s. l. are generally variable between different clades. Non-lichenicolous species mainly exhibit jelly-like basidiomata with cerebriform, folise, lobe, or pulvinate macromorphology and white, yellow, orange, or brown colour. In addition, some species are intrahymenial parasites that occur within the hymenia of dacrymycetaceous or corticoid species. Their basidiomata are not macroscopically visible. Lichenicolous species usually produce inconspicuous gall deformations on the thallus of lichens, at least in early stages of growth, where as some species can induce the formation of large galls up to 15 mm in diameter (Diederich 1996, 2007). Some species can produce gelatinous basidiomata instead of gall formation (Diederich 1996; Lindgren et al. 2015; Millanes et al. 2015; Zamora et al. 2017). Moreover, some species grow intrahymenially without any external symptoms (Diederich 1996, 2007). Compared to the increasing knowledge of the diversity of lichenicolous species, few studies of non-lichenicolous *Tremella* species are published in recent years.

*Tremella* s. s. is now confined to Fuciformis and Mesenterica subclades containing more than 30 species. Basidiomata of some *Tremella* s. s. species have long been used as food or traditional medicine in China or other Asian countries. *Tremellafuciformis* and *T.aurantialba* have been cultivated in China for more than 30 years. The diversity and distribution of *Tremella* are poorly known in China, as comparatively few mycologists focus on these fungi (Peng 1982; Bandoni and Zang 1990). In the present study, four new species are described and characterised based on morphological characters and phylogenetic analyses of nuc rDNA ITS region and nuc 28S rDNA D1/D2 domain.

## Materials and methods

### Sampling and morphological examination

Specimens were collected from Guangdong, Guangxi, Heilongjiang, Jilin, Qinghai, Tibet, and Yunnan provinces in China. The specimens were air dried immediately after their collection. Macromorphological descriptions were based on field observations. Micromorphological examination followed the studies by Chen (1998) and Millanes et al. (2014). Microscopic structures, features, and measurements were observed using handmade sections stained with 1% Phloxin after pretreatment with 5% KOH and photographed with Zeiss AXIO Imager A2 coupled to an AxioCam MRc5 digital camera. Basidiospores and conidia measurements are present as follows: length range × width range, L = mean spore length (arithmetic average of all spores), W = mean spore width (arithmetic average of all spores), Q = variation in the L/W ratios and *n* = number of spores measured. All specimens were preserved in the XZL culture collection (personal culture collection of Xin-zhan Liu housed in the Institute of Microbiology, Chinese Academy of Sciences). Type specimens were deposited in Mycological Herbarium of the Institute of Microbiology, Chinese Academy of Sciences, Beijing, China (HMAS). The cultures were deposited in China General Microbiological Culture Collection Center (CGMCC) and the CBS yeast collection of the Westerdijk Fungal Biodiversity Institute, Utrecht, the Netherlands.

### DNA extraction, PCR amplification and sequencing

DNA was extracted directly from the specimens examined. A very small amount of dry tissue was soaked in sterile water for 30 min and dried with sterile filter papers. The tissue was taken into 2 ml eppendorf tube with quartz sand (1–2 mm), lyophilized using liquid nitrogen and immediately crushed with tissue grinder for 2 min using SCIENTZ-48 at 70 Hz (SCIENTZ, China). The sample was homogenized in 1 ml 5% CTAB preheated at 65 °C. The mixture was warmed up at 65 °C for 1 h and centrifuged by 15000 rpm for 15 min. The supernatant was purified with phenol:chloroform:isoamyl alcohol (25:24:1) for twice of which the second purification step without phenol. The supernatant was incubated for 30 min at 37 °C with 25 μl RNAase (20 mg/ml) and then purified again. The precipitation with 3 M sodium acetate and ethyl alcohol absolute was conducted. Finally, the DNA was washed twice with 70% (w/v) ethanol and then dissolved in 50 μl of pure water. The nuc rDNA ITS region and D1/D2 domain of nuc 28S rDNA were amplified using the protocols described previously (Liu et al. 2015a). PCR products were observed on 1% Agarose gel electrophoresis stained with ethidium bromide. Purification and sequencing of PCR products were carried out at TSINGKE Biological Technology, Beijing, China.

### Phylogenetic analyses

Phylogenetic analyses were performed as described previously with modification (Millanes et al. 2011; Liu et al. 2015a, b). *Vishniacozymacarnescens* CBS 973^T^ was chosen as outgroup because the genera *Vishniacozyma* is the sister group of Tremellaceae (Liu et al. 2015a, b). Four partitions, i.e., ITS1, 5.8S, ITS2 and D1/D2 domain, were chosen as the appropriate scheme (Millanes et al. 2011; Zamora et al. 2017). Multiple sequences were aligned using MAFFT algorithm and the G-INS-I option (Standley 2013). Major insertions and ambiguous regions were identified and eliminated with Gblocks version 0.91b (Castresana 2000) using a relaxed selection (minimum number of sequences for a conserved position = 36, minimum number of sequences for a flank position = 60, maximum number of contiguous non-conserved positions = 10, minimum length of a block = 5 and allowed gap positions = ‘with half’), following Talavera and Castresana (2007). PartitionFinder V2.1.1 (Lanfear et al. 2017) was used to determine the best-fit evolutionary model for each partition, with the following settings: the ‘all’ search algorithm, the corrected Akaike Information Criterion (AICc) for model selection and either the ‘raxml’ or ‘mrbayes’ set of models.

Dataset congruence was assessed manually by analyzing the datasets separately by maximum likelihood bootstrapping. Conflict among clades was considered significant if a significantly supported clade (bootstrap support ≥ 70%; Hillis and Bull 1993) for one marker was contradicted with significantly supported by another. Incongruence was found between topologies derived from ITS1, 5.8S, ITS2, and D1/D2 domain.

Maximum likelihood (ML) analyses of single gene were performed in RAxML-HPC V.8 (Stamatakis 2014) on the CIPRES Science Gateway (Miller et al. 2010). The GTR+G, GTR+G, GTR+I+G and GTR+I+G models were applied to each partition. The best-scoring tree was obtained using rapid bootstrap analysis by running 1000 replicates. Four single-gene trees estimated above were then used as input to infer the species tree with the coalescent-based approach implemented in the ASTRAL program v5.6.3 (Mirarab and Warnow 2015). The bootstrapping option of ASTRAL was used for 1000 replicates.

Bayesian analyses were conducted by Markov Chain Monte Carlo (MCMC) sampling for combined nucleotide sequences using MRBAYES 3.2.2 (Ronquist et al. 2012) on the CIPRES Science Gateway (Miller et al. 2010). Likelihood models were selected for each of the four gene partitions among the 24 models implemented in MrBayes. A HKY+I+G model was selected for the ITS1, a K80+G model was selected for the 5.8S, a SYM+I+G was selected for the ITS2 and a GTR+I+G model was selected for D1/D2 domain. Two independent runs were executed, each with four chains, three of which were incrementally heated. The analysis was conducted for 5 million generations with trees sampled every 5000 generations. The first 25% trees, which represent the burn-in phase of the analysis, were discarded after checking for stability on the log-likelihood curves and the split-frequencies of the runs in Tracer v.1.7 (Rambaut et al. 2018). The remaining trees were used for calculating posterior probabilities (PP) in the majority rule consensus tree.

Branches that received bootstrap values (BP) for Maximum likelihood and Bayesian posterior probabilities (BPP) greater than or equal to 50% (BP) and 0.95 (BPP) were considered as significantly supported. The GenBank accession numbers for the sequences of the ITS region and D1/D2 domain used in this study are listed in Table 1.

**Table 1.** Sequences used in molecular phylogentic analysis. Entries in bold are newly generated for this study.

Species	Strain number	Voucher number	Country	ITS	D1D2
*** Tremella basidiomaticola ***	**CGMCC 2.5724^T^**	–	**China, Fujian**	**MH712820**	**MH712784**
	**CGMCC 2.5725**	–	**China, Fujian**	**MH712821**	**MH712785**
	**CBS 8225**	–	**China, Fujian**	**MH712822**	**MH712786**
* Tremella brasiliensis *	CBS 6966^R^	–	Costa Rica	AF444429	AF189864
	CBS 8231	–	Costa Rica	JN053465	JN043570
* Tremella cerebriformis *	–	LE 296436	Russia	KP986538	/
	–	LE 303455	Russia	KP986522	/
	–	VLA M-11693	Russia	KP986538	/
*** Tremella cerebriformis ***	–	**ZRL20170101**	**China, Heilongjiang**	**MH712823**	**MH712787**
	–	**ZRL2017026**9	**China, Heilongjiang**	**MH712824**	**MH712788**
*** Tremella cheejenii ***	–	**GX20172598**	**China, Guangxi**	**MH712825**	**MH712789**
	–	**GX20172640**	**China, Guangxi**	**MH712826**	**MH712790**
* Tremella dysenterica *	–	LE 303447	Russia	KP986509	KP986542
	–	VLA M-18599	Russia	KP986531	/
*** Tremella erythrina ***	–	**GX20170141 (HMAS 255317)**	**China, Guangxi**	**MH712827**	**MH712791**
	–	**GX20170916001 (HMAS 279591)**	**China, Guangxi**	**MH712828**	**MH712792**
* Tremella fibulifera *	–	LE 303445	Russia	KP986518	KP986547
*** Tremella fibulifera ***	–	**GX20172028**	**China, Guangxi**	**MH712829**	**MH712793**
	–	**HMAS 5285**2	**China, Tibet**	**MH712830**	**MH712794**
* Tremella flava *	CBS 8471^R^	–	Taiwan	KY105681	KY105681
	–	CCJ 907	Taiwan	AF042221	AF042403
	–	CCJ 928	Taiwan	AF042223	AF042405
*** Tremella flava ***	–	**ZRL20180289**	**China, Yunnan**	**MH712834**	**MH712798**
	–	**ZRL20180156**	**China, Yunnan**	**MH712835**	**MH712799**
	–	**KM20170128**	**China, Yunnan**	**MH712836**	**MH712800**
	–	**YN135**	**China, Yunnan**	**MH712837**	**MH712801**
	–	**ZRL20180167**	**China, Yunnan**	**MH712838**	**MH712802**
	–	**ZRL20180164**	**China, Yunnan**	**MH712839**	**MH712803**
	–	**ZRL20180166**	**China, Yunnan**	**MH712840**	**MH712804**
	–	**ZRL20180348**	**China, Yunnan**	**MH712841**	**MH712805**
	–	**ZRL20180349**	**China, Yunnan**	**MH712842**	**MH712806**
	–	**23**	**China, Yunnan**	**MH712843**	**MH712807**
	–	**24**	**China, Yunnan**	**MH712844**	**MH712808**
	–	**YN177**	**China, Yunnan**	**MH712845**	**MH712809**
	–	**YN180**	**China, Yunnan**	**MH712846**	**MH712810**
* Tremella fuciformis *	CBS 6970^R^		Taiwan	KY105683	AF075476
	–	CCJ 1072	Taiwan	AF042227	AF042409
	–	CCJ 1531	Taiwan	AF042254	AF042436
*** Tremella fuciformis ***	–	**GX20170212**	**China, Guangxi**	**MH712831**	**MH712795**
	–	**GX20172644**	**China, Guangxi**	**MH712832**	**MH712796**
	–	**HMAS 0274334**	**China, Tibet**	**MH712833**	**MH712797**
* Tremella fuciformis *	CBS 6971	–	USA	KY105682	KY109896
* Tremella globispora *	CBS 6972^R^	–	Canada	AF444432	AF189869
	–	UBC 586	Canada	AF042425	AF042243
* Tremella laurisilvae *	–	Koschatzky s.n.	Portugal	JN053467	JN043572
* Tremella lloydiae-candidae *	–	VLA M-11702	Russia	KP986536	KP986559
	–	VLA M-11703	Russia	KP986537	KP986560
* Tremella mesenterica *	CBS 6973^R^	–	Canada	AF444433	AF075518
	–	Ryman 9146	Sweden	JN053463	JN043568
	–	CCJ 1040	Taiwan	AF042408	AF042226
	–	FO 24610	German	AF042447	AF042265
*** Tremella mesenterica ***	–	**HMAS 270832**	**China, Guangdong**	**MH712847**	**MH712811**
	–	**HMAS 88438**	**China, Jilin**	**MH712848**	**MH712812**
	–	**HMAS 96841**	**China, Qinghai**	**MH712849**	**MH712813**
	–	**GX20170708**	**China, Guangxi**	**MH712850**	**MH712814**
* Tremella resupinata *	–	CCJ 1458	Taiwan	AF042421	AF042239
*** Tremella salmonea ***	–	**GX20172637**	**China, Guangxi**	**MH712851**	**MH712815**
* Tremella samoensis *	–	LE 262897	Russia	KP986511	/
	–	VLA M-18603	Russia	KP986532	KP986555
*** Tremella samoensis ***	–	**GX20172371**	**China, Guangxi**	**MH712852**	**MH712816**
	–	**GX20170536**	**China, Guangxi**	**MH712853**	**MH712817**
* Tremella taiwanensis *	–	CCJ 1151	Taiwan	AF042412	AF042230
	–	CCJ 1153	Taiwan	AF042413	AF042231
*** Tremella taiwanensis ***	–	**GX20170625**	**China, Guangxi**	**MH712854**	**MH712818**
	–	**GX20170629**	**China, Guangxi**	**MH712855**	**MH712819**
* Tremella tropica *	CBS 8483^R^	–	Taiwan	KY105697	KY109908
	CBS 8486	–	Taiwan	KY105697	KY109909
	–	CCJ 1355	Taiwan	AF042433	AF042251
* Tremella yokohamensis *	JCM 16989^T^	–	Japan	HM222926	HM222927
	–	VLA M-11700	Russia	KP986529	/
Outgroup	–	–	–	–	–
* Cryptococcus depauperatus *	CBS 7841^T^	–	–	FJ534881	FJ534911

## Results

### Phylogenetic analyses

**Figure 1.**
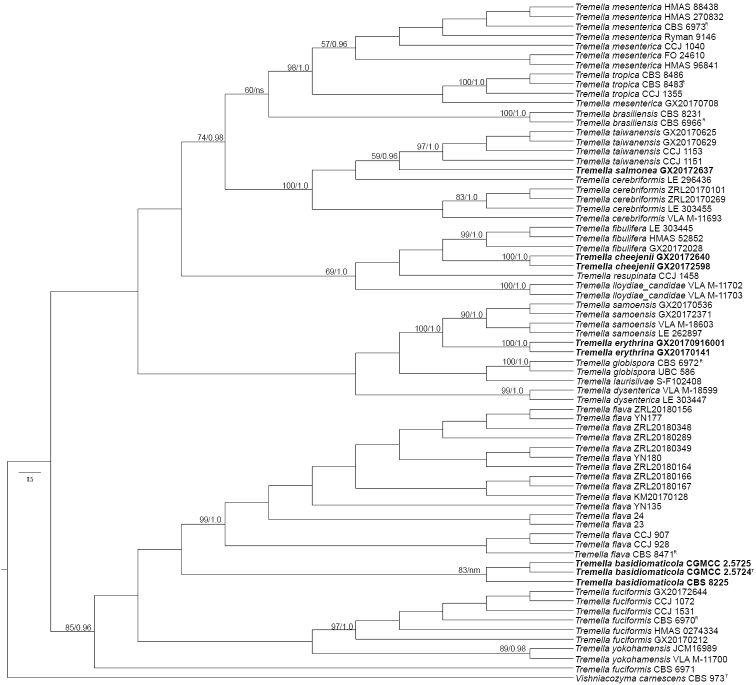
The maximum likelihood tree of the novel species and related taxa in *Tremella* sensu stricto based on the combined sequences of the nuc rDNA ITS region and nuc 28S rDNA D1/D2 domain. Bootstrap supports (BP) (> 50%) of maximum likelihood method and Bayesian posterior probability (BPP) values (> 0.9) are shown at each node. Note: ns, not supported (BP < 50% or PP < 0.9); nm, not monophyletic.

The combined dataset consisted of ITS1 region (44 bp), 5.8S region (156 bp), ITS2 region (168 bp), and D1/D2 domain (532 bp) (a total of 900 bp) for 57 specimens and 13 strains in genus *Tremella* with *Vishniacozymacarnescens* CBS 973^T^ as the outgroup. Two methods for phylogenetic tree construction resulted in a similar topology. Therefore, only the best scoring RAxML tree is shown with BP and BPP values simultaneously in Figure 1. All the *Tremella* specimens and strains in this study separated into 19 clades, representing 15 known and four new species. The four new species clustered into distinct clades supported with high bootstrap values.

### Taxonomy

#### Tremella basidiomaticola

Taxon classificationFungiTremellalesTremellaceae

X.Z. Liu & F.Y. Bai sp. nov.

MycoBank: MB827184

Figure 2

##### Type.

CHINA, Fujian Province, Ningde city, Gutian county, on the basidioma of *Tremellafuciformis*, July 2017, X.Z. Liu (holotype strain: CGMCC 2.5724^T^, ex-holotype strain: CBS 15261^T^).

##### Etymology.

*Basidiomaticola* refers to the species isolated from the basidioma of *T.fuciformis*.

##### Description.

**Asexual morph**: colonies yellowish, smooth, shiny, and slimy, with an entire margin. Pseudohyphae and hyphae are not formed on corn meal agar. Conidia hyaline, smooth, globose to subglobose, 3.0–6.0 × 2.5–5.0 μm, L = 4.8 ± 0.9 μm, W = 3.9 ± 0.8 μm, Q = 1.0–1.7 (*n* = 30). Ballistoconidia, globose to subglobose on CMA agar, 5.0–7.0 × 3.5–6.0 μm, L = 6.0 ± 0.6 μm, W = 5.1 ± 0. 6 μm (*n* = 30). The comparison of physiological properties between this new species and its related taxa were listed in Suppl. material 1. **Sexual morph**: undetermined.

**Figure 2.**
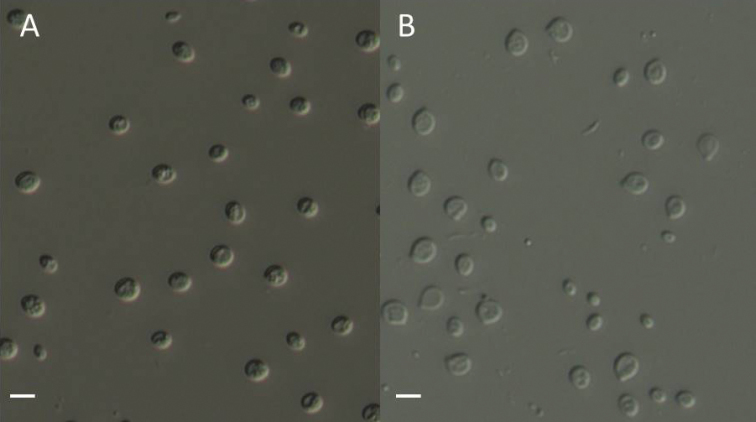
*Tremellabasidiomaticola*CGMCC 2.5724^T^**A** Vegetative cells grown in YM agar for 7 days at 17 °C **B B**allistoconidia produced on CMA agar for 7 days at 17 °C. Scale bars: 5 μm.

##### Additional isolate examined.

CHINA, Fujian Province, on the basidioma of *Tremellafuciformis*, July 2017, X.Z. Liu, CGMCC 2.5725 = CBS 15262; Japan, isolated from Mori Ind. Co., Ltd, 1968, T. Suda, NBRC 8990 = CBS 8225.

##### Notes.

Three strains representing *T.basidiomaticola* clustered in a well-supported clade that closely related to *T.yokohamensis*, *T.flava*, and *T.fuciformis*. *Tremellabasidiomaticola*CGMCC 2.5724^T^ differed from *T.yokohamensis*, *T.flava*, and *T.fuciformis* by 97.4%, 94.4%–95.1%, and 97.8%–98.1% sequence identities in D1/D2 domain and 96.3%–96.6%, 94.4%–95.7%, and 96.6%–97.5% sequence identities in ITS region. Physiologically, the ability to assimilate lactose, melibiose, raffinose, inulin, soluble starch, L-rhamnose, ethanol, glycerol, DL-lactic acid, and inositol were different between *T.basidiomaticola* and closely related taxa (Suppl. material 1: Table S1). Moreover, the novel species can grow in vitamin-free medium but not for its sister species.

#### Tremella cheejenii

Taxon classificationFungiTremellalesTremellaceae

X.Z. Liu & F.Y. Bai sp. nov.

MycoBank: MB827187

Figures 3 , 4

##### Type.

CHINA. Guangxi Province, Hechi city, Luocheng county, Pingying village, Jiuwan Mountain National Nature Reserve, 108°48'E, 25°19'N, G.J. Li, H.S. Ma, Z.L. Lin & M.Z. Zhang, 7 August 2017, GX20172598 (HMAS 279589).

##### Etymology.

*Cheejenii* was named in honor of Chee-Jen Chen for his contributions to systematics of tremellalean fungi.

##### Description.

Basidiomata sessile, cerebriform, up to 1.0–3.0 cm in diameter, broadly attached to substratum, soft gelatinous, pale white when fresh and pale brown in dry condition. Hyphae smooth, thick-walled, slender, 2.0–4.5 μm in diameter, often anastomosing, clamp connections abundant, loop-like forming a large hollow. Haustoria rare, small, subglobose, ca 2.0 μm in diameter, with a single hypha. Hyphidia abundant, smooth, thin-walled, 2.5–4.0 μm in diameter, branched, hyphidia and basidia derived from the same hypha. Probasidial initials subglobose, ovoid or pyriform. Mature basidia subglobose, broadly ellipsoid or ovoid, mostly two-celled, and occasionally four-celled, with apical protuberance, often longitudinally septate or occasionally oblique or cruciate-septate, thin-walled, 12.0–17.0 μm × 13.0–18.0 μm, stalked, 2.0–4.0 μm long, with sterigmata up to 70 μm, not swollen at apex. Basidiospores hyaline, smooth, thin-walled, subglobose to broadly ellipsoid, apiculate, 5.0–10.0 μm × 4.5–8.0 μm, L = 8.6 ± 1.1 μm, W = 6.6 ± 0.8 μm, Q = 1.1–1.8 (*n* = 40). Basidiospores forming secondary ballistoconidia by the formation of a sterigma. Conidia ellipsoid, smooth, hyaline, thin-walled, 2.2–4.0 μm × 1.8–3.0 μm, L = 3.1 ± 0.6 μm, W = 2.2 ± 0.3 μm, Q = 1.0–2.0 (*n* = 40), monokaryotic, budding from apex of sterigmata.

**Figure 3.**
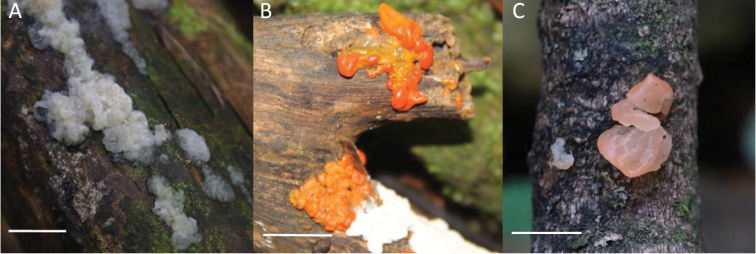
Macromorphology of *Tremella* basidiomata. **A***T.cheejenii***B***T.erythrina* C *T.salmonea*. Scale bars: 1 cm.

**Figure 4.**
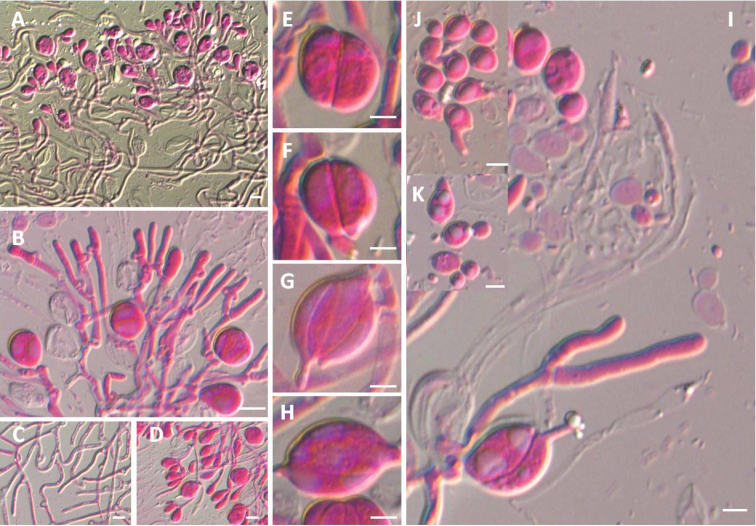
Microscopic structure of *Tremellacheejenii* (HMAS 279589). **A** Section through hymenium **B** Hyphidia from context **C** Hyphae from context **D** Probasidia **E–H** Mature basidia **I** Mature basidia and conidia produced from the sterigmata **J–K** Basidiospres and its germination with short sterigma. Scale bars: 10 μm (**A–D**), 5 μm (**E–J**).

##### Habitat.

On wood of deciduous tree, in forest dominated by Fagaceae, Lauraceae, Theaceae, Magnoliaceae, and Hamamelidaceae.

##### Additional specimens examined.

CHINA. Guangxi Province, Hechi city, Luocheng county, Pingying village, Jiuwan Mountain National Nature Reserve, 108°48'E, 25°19'N, G.J. Li, H.S. Ma, Z.L. Lin & M.Z. Zhang, 7 August 2017, GX20172640 (HMAS 279590).

##### Notes.

Two specimens form the sister group to *T.fibulifera*, *T.lloydiae-candidae*, and *T.resupinata* and represent a new species, *T.cheejenii*. The sequence identities between *T.cheejenii* and *T.fibulifera* are 95.7%–95.9% and 92.5%–93.2% in the D1/D2 domain and ITS region, respectively. Similarly, *T.cheejenii* and *T.lloydiae-candidae* showed 96.1%–96.2% and 92.1% sequence identities in the D1/D2 domain and ITS region, respectively. *Tremellacheejenii* and *T.resupinata* showed 90.4% and 89.9% sequence identities in the D1/D2 domain and ITS region, respectively. *Tremellacheejenii* is distinct from *T.fibulifera* in its bigger basidia (12.0–17.0 μm × 13.0–18.0 μm in *T.cheejenii* vs 14–16 μm × 10–13 μm in *T.fibulifera*). However, the basidia of *T.cheejenii* are smaller than that of *T.resupinata* (12.0–17.0 μm × 13.0–18.0 μm in *T.cheejenii* vs 27.0–40.0 μm × 22.0–31.0 μm in *T.resupinata*) (Chen 1998; Malysheva et al. 2015). Moreover, conidia are produced from the sterigmata in *T.cheejenii* compared to the absence of conidia in *T.fibulifera*, *T.lloydiae-candidae*, and *T.resupinata*.

#### Tremella erythrina

Taxon classificationFungiTremellalesTremellaceae

X.Z. Liu & F.Y. Bai sp. nov.

MycoBank: MB827186

Figures 3 , 5

##### Type.

CHINA. Guangxi Province, Chongzuo city, Longzhou county, Qiang village, Nonggang National Nature Reserve, 106°54'E, 22°27'N, R.L. Zhao, M.Q. He, G.F. Mou, J.L. Qin, H.J. Wang & X.Y. Zhu, 30 July 2017, GX20170141 (HMAS 255317).

##### Etymology.

*Erythrina* refers to the colour of the basidioma.

##### Description.

Basidiomata sessile, cerebriform to foliose, with undulate broad lobes, lobes hollow, firm gelatinous, up to 1.3–1.8 cm in diameter, broadly attached to substrate, red and brownish orange when fresh and brownish orange when dry. Hyphae smooth, thin- or thick-walled, slender, hyaline, 1.0–3.0 μm, with clamp connections, branched with frequent anastomoses. Haustoria rare, small, subglobose, 1.5–2.0 μm in diameter, with single hyphae. Hyphidia present, smooth, thin-walled, 2.0–4.0 μm, branched. Probasidia mostly broadly ellipsoid. Mature basidia, globose to subglobose or broadly ellipsoid to ovoid, 12.0–18.0 μm × 13.0–19.0 μm, mostly four-celled, occasionally two-celled, without stalks, frenquently longitudianllly cruciate-septate. Basidiospores, smooth, thin-walled, ellipsoid to ovoid, apiculate, 7.0–10.0 μm × 5.0–7.0 μm, L = 8.2 ± 0.8 μm, W = 6.1 ± 0.6 μm, Q = 1.1–1.7 (*n* = 40).

**Figure 5.**
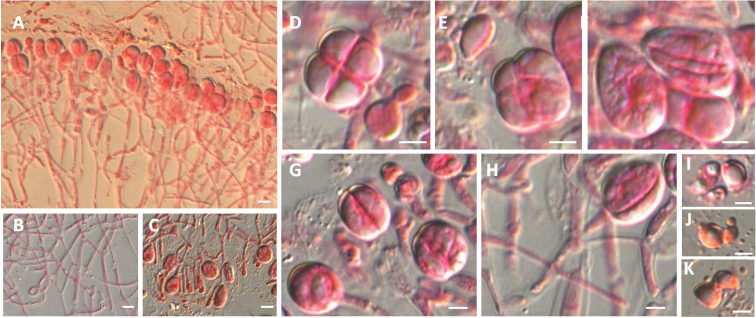
Microscopic structure of *Tremellaerythrina* (HMAS 255317). **A** Section through hymenium **B** Hyphae from context **C** Hyphidia with basidia of different developmental stages **D–H** Mature basidia **I–K** Basidiospres. Scale bars: 10 μm (**A–C**), 5 μm (**D–K**).

##### Habitat.

On decaying wood of deciduous tree, in forest dominated by Anacardiaceae, Palmae, Hypericaceae, and Sterculiaceae.

##### Additional specimens examined.

CHINA. Guangxi Province, Chongzuo city, Longzhou county, Nonggang village, Nonggang National Nature Reserve, 106°56'E, 22°28'N, H.S. Ma, 16 September 2017, GX20170916001 (HMAS 279591).

##### Notes.

Two specimens representing *T.erythrina* clustered in a well-supported clade and were closely related to *T.samoensis*. These two species showed 97.6%–97.8% and 93.7%–96.0% sequence identities in the D1/D2 domain and ITS region, respectively. Basidia in *T.erythrina* are larger than those of *T.samoensis* (12.0–18.0 μm × 13.0–19.0 μm in *T.erythrina* vs 12.0–18.0 μm × 8.0–12.0 μm in *T.samoensis*) (Chen 1998; Malysheva et al. 2015). Moreover, hyphidia are present and located in the hymenial structure and derived from the same generative hyphae with basidia in *T.erythrina*, whereas hyphidia are lacking in *T.samoensis* (Chen 1998; Malysheva et al. 2015).

#### Tremella salmonea

Taxon classificationFungiTremellalesTremellaceae

X.Z. Liu & F.Y. Bai sp. nov.

MycoBank: MB827188

Figures 3 , 6

##### Type.

CHINA. Guangxi Province, Hechi city, Luocheng county, Jiuwan Mountain National Nature Reserve, 108°48'E, 25°19'N, G.J. Li, H.S. Ma, Z.L. Lin & M.Z. Zhang, 7 August 2017, GX20172637 (HMAS 279588).

##### Etymology.

*Salmonea* refers to the colour of the basidioma.

##### Description.

Basidiomata small, gyrose to cerebriform, 0.6–1.0 cm in diameter, firm gelatinous and thick, pale orange when fresh, yellow orange when dry, flat on the substrate. Hyphae smooth, thin-walled, slender, 2.0–3.5 μm in diameter, often with clamp connections. Haustoria rare, small, globose or subglobose, 2.0–4.0 μm in diameter, with single hyphae. Hyphidia rare, smooth, thin-walled, 2.0–4.0 μm, branched. Probasidial initials mostly subglobose to globose, sometimes broadly ellipsoid. Basidia, when mature, subglobose to globose, four-celled, occasionally two-celled, thin-walled, 31.0–38.0 μm × 29.0–37.0 μm, with longitudinally cruciate-septate, without stalk-like base; sterigmata up to 110.0 μm long, not swollen at apex. Basidiospores globose to subglobose, 16.0–22.0 μm × 15–20.0 μm, L = 18.3 ± 1.3 μm, W = 17.8 ± 1.4 μm, Q = 0.9–1.3 (*n* = 25), with a distinct apiculus. Conidia present, ellipsoid, fusiform to cylindrical, 8.0–17.0 μm × 2.0–5.0 μm, L = 10.7 ± 2.2 μm, W = 3.5 ± 0.5 μm, Q = 2–5.8 (*n* = 40), hyaline, clamped, arranged in cluster. Terminally and laterally swollen cells appearing abundant in the subhymenium, citriniform, pyriform or broadly ellipsoid, 9.0–20.0 μm × 5.6–13.0 μm, L = 14.2 ± 2.8 μm, W = 8.8 ± 1.8 μm, Q = 1.1–2.8 (*n* = 40).

**Figure 6.**
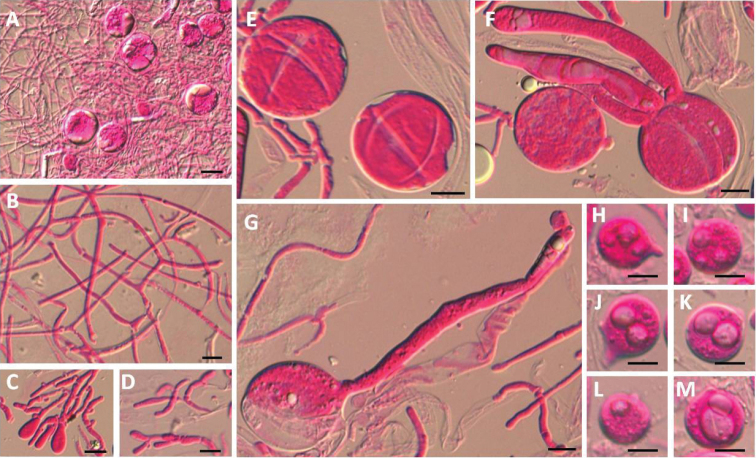
Microscopic structure of *Tremellasalmonea* (HMAS 279588). **A** Section through hymenium **B** Hyphae from context **C** Swollen cells **D** Conidia in cluster **E–G** Mature basidia **H–M** Basidiospres. Scale bars: 10 μm (**A–M**).

##### Habitat.

On wood of deciduous tree, in forest dominated by Rosaceae, Moraceae, Lauraceae, and Theaceae.

##### Notes.

Only one specimen representing *T.salmonea* formed a distinct clade closely related to *T.taiwanensis* with 96.8%–98.3% sequence identities in D1/D2 domain and 95.4%–96.6% in ITS region, respectively. The affinity of *T.salmonea* to *T.taiwanensis*lacked high support by the coalescent-based method (Fig. 1). *Tremellasalmonea* differs from *T.taiwanensis* in its larger basidia (31.0–38.0 μm × 29.0–37.0 μm in *T.salmonea* vs 23.0–29.0 μm × 22.0–27.0 μm in *T.taiwanensis*) and basidiospores (16.0–22.0 μm × 15.0–20.0 μm in *T.salmonea* vs 14.0–18.0 μm ×14.0–20.0 μm in *T.taiwanensis*). In addition, hyphae-like conidiogenous cells and dikaryotic conidia were observed in *T.salmonea* compared to monokaryotic conidia produced from apex of sterigmata (Chen 1998). Swollen cells were located in the hymenium in *T.salmonea* whereas they were absent in *T.taiwanensis* (Chen 1998).

## Discussion

*Tremella* s. s. is characterized by their tremella-like basidiomata. Many morphological characteristics have been used in taxonomic studies of *Tremella*, including the shape, colour, and size of basidiomata, basidia, and basidiospores, as well as other features such as length of the stalks and sterigmata, spore formation of the basidia, conidia, swollen cells, and hyphidia (Chen 1998). However, morphology-based taxonomy of *Tremella* species is very complicated. Almost 30 macromorphological and micromorphological characters need to be checked for identification at the species level (Chen 1998). Morphological taxonomy cannot provide enough evidence of phylogenetic relationship. Morphologically, *Tremellaglobispora* resembles species in the Indercorata group by its pyriform to capitates basidia and its spores that are broader than long (Chen 1998). Nevertheless, it is more related to species in the Fuciformis group, based on molecular data (Chen 1998; Fell et al. 2000; Scorzetti et al. 2002; Liu et al. 2015b). The application of molecular phylogenetics has significantly benefited the systematics and taxonomy of *Tremella*. In the present study, four new species of genus *Tremella* are described from China based on both morphological and molecular data.

The fruiting bodies of fungi harbour diverse microbial community including bacteria, yeasts and filamentous fungi (Buzzini et al. 2005; Barbieri et al. 2007; Pacioni et al. 2007). Microbial habitants could have roles in the development of the fruiting body, such as mycelium growth, nutrition supply, antifungal activity, and flavour formation (Sbrana et al. 2002; Barbieri et al. 2007; Antony-Babu et al. 2013; Seung-Yoon et al. 2018). There have been a new bacterial species found in the fruiting body of *T.fuciformis* which can cause infection (Wen et al. 2016). *Tremellabasidiomaticola* was isolated from the fruit body of *T.fuciformis* and their relationship and contributions to the growth of fruiting body remain unknown.

*Tremellasalmonea* is highly supported as belonging to the Mesenterica group. Microscopically, *T.salmonea* and *T.mesenterica* are similar in that both species share loose a hymenial structure with abundant hyphidia. However, these two species have different basidiomata colour: in *T.salmonea* basidiomata are salmon-orange, whereas in *T.mesenterica* they are yellowish. Other species in the *T.mesenterica* group with similar basidiomata colour include *T.roseolutescens* (basidia 20–27 μm × 18–27 μm) and *T.tropica*(basidia 19–21 μm × 15–17 μm), but these are clearly different in the shape of their basidiomata and size of their basidia (Bandoni et al. 1996; Chen 1998; Roberts 2008).

The affiliation of *T.cheejenii* and *T.erythrina* to the Fuciformis or Mesenterica groups were not ascertained phylogenetically. *Tremellacheejenii* are closely related to T. fibulife*ra*, *T.lloydiae-candidae*, and *T.resupinata* in the phylogenetic analysis. Though they all have white basidiomata, there are clear differences in the shape and size of their basidiamata, length of their basidia and stalks, and length of their sterigmata (Bandoni and Oberwinkler 1983; Chen 1998; Malysheva et al. 2015). *Tremellahainanensis* also has whitish basidiomata, but it is distinguished from *T.cheejenii* by its filamentous lobes and ball-like basidiomata (Peng 1982). *Tremellaerythrina* is closely related to *T.samoensis*, nevertheless, *T.erythrina* is distinguished by its salmon-orange cerebriform basidiomata that are larger than in *T.samoensis* (Chen 1998). Macroscopically, the most similar species to *T.erythrina* is *T.armeniaca*, *T.elastica*, *T.roseolutescens*, and *T.tawa*, all of which have orange basidiomata. *Tremellaroseolutescens* (basidia 20–27 μm × 18–27 μm; basidiospores 11–15 μm × 9–11.5 μm) is diagnosed by its pulvinate basidiomata and larger basidia and basidiospores differing from *T.erythrina* (basidia 12–18 μm × 13–19 μm; basidiospores 7–10 μm × 5–7 μm) (Bandoni et al. 1996). Basidia in *T.erythrina* are slightly larger than those of *T.elastic* (10.0–15.0 μm × 6.0–9.0 μm) (Chen 1998). The presence of conidia and phialide-like conidiogenous cells in the hymenium of *T.armeniaca* has not been discovered in *T.erythrina* (Bandoni et al. 1996). *Tremellatawa* (basidia 20–30 μm × 13–18 μm) differs from *T.erythrina* in its clavate basidia and larger basidiomata and basidia (Bandoni and Buchanan 1990).

A total of 33 specimens of *Tremella* s. s. were collected from seven provinces (Guangdong, Guangxi, Heilongjiang, Jilin, Qinghai, Tibet, and Yunnan), which span a large portion of China and have different climates, humidity, and vegetation types. This implies the genus is really diverse beyond current knowledge. *Tremella* s. s. showed a significant deviation from the optimal range calculated for the genus rank using the phylogenetic rank boundary optimization (RPBO) analysis that indicates great genetic variation between different species in *Tremella* s. s. (Liu et al. 2015b). Two subclades, namely Mesenterica and Fuciformis, are included in this genus and can be featured by distinct ecological and morphological characters (Chen 1998, Liu et al. 2015b). They could probably be reclassified as two separate genera in the future. Further studies with additional fresh collections will clarify the systematic of this genus and enrich the knowledge of distribution, abundance, and ecology of *Tremella* species.

### Key to the whitish species in *Tremella* s. s.

**Table:** 

1	Basidia with sterigmata shorter than 35, hyphae grow from side of hyphae	**2**
–	Basidia with sterigmata longer than 35, hyphae grow from basidial clamp	**3**
2	Basidiomata gyrose to cerebriform, 1–3 cm in diameter and basidia > 10 μm long	*** T. lloydiae-candidae ***
–	Basidiomata foliose, larger than 3 cm in diameter and basidia < 10 μm long	**4**
3	Basidiomata filamentous lobes, conjunctive as a ball	*** T. hainanensis ***
–	Basidiomata resupinate or gyrose to cerebriform	**5**
4	Basidia globose to subglobose	*** T. fuciformis ***
–	Basidia clavate with stalks	*** T. yokohamensis ***
5	Basidiospores mostly broader than long	*** T. globispora ***
–	Basidiospores mostly longer than broad	**6**
6	Basidiomata resupinate, < 1 cm in diameter	*** T. resupinata ***
–	Basidiomata gyrose to cerebriform, usually > 1 cm in diameter	**7**
7	Basidia size longer than 30 μm and basidiospores > 17 μm long	*** T. cerebriformis ***
–	Basidia size smaller than 20 μm and basidiospores ≤ 10 μm in long	**8**
8	Basidia > 13 μm wide, with short stalk, sterigmata with inconspicuous apically swollen	*** T. cheejenii ***
–	Basidia < 13μm wide, without stalk, sterigmata with conspicuous apically swollen	*** T. fibulifera ***

### Key to the yellow, orange, or red species in *Tremella* s. s.

**Table:** 

1	Basidiomata yellow	**2**
–	Basidiomata orange or red	**11**
2	Basidia mostly > 25 μm long	**3**
–	Basidia mostly < 25 μm long	**4**
3	Basidia < 22 μm wide and basidiospores 10–12 μm long	*** T. philippinensis ***
–	Basidia > 26 μm wide and basidiospores > 13 μm long	*** T. brasiliensis ***
4	Basidiomata pulvinate	*** T. subrubiginosa ***
–	Basidiomata gyrose to cerebriform or foliose	**5**
5	Basidiomata gyrose to cerebriform	**6**
–	Basidiomata foliose	**8**
6	Vesicles absent, haustoria rare, and conidia monokaryotic budding from apex of sterigmata	*** T. taiwanensis ***
–	Vesicles present, haustoria abundant, and conidia dikaryotic from hyphae-like conidiogenous cells	**7**
7	Basidiospores broadly ellipsoid or ovoid	*** T. mesenterica ***
–	Basidiospores globose to subglobose	*** T. mesenterella ***
8	Basidia > 17 μm long and basidiospores > 7 μm wide	*** T. iduensis ***
–	Basidia < 17 μm long and basidiospores < 7 μm wide	**9**
9	Basidiomata lobes not hollow	*** T. boninensis ***
–	Basidiomata lobes hollow	**10**
10	Haustoria abundant and branched, probasidia mostly growing from side of the hymenial hyphae	*** T. flava ***
–	Haustoria rare, probasidia proliferating directly from basidial clamps	*** T. samoensis ***
11	Basidiomata pulvinate	*** T. roseolutescens ***
–	Basidiomata gyrose to cerebriform or foliose	**12**
12	Basidiomata foliose and flat; basidia > 30 μm long	*** T. salmonea ***
–	Basidiomata gyrose to cerebriform; basidia < 30 μm long	**13**
13	Basidiomata reddish	**14**
–	Basidiomata orange	**15**
14	Basidia 17–21 μm long	*** T. rubromaculata ***
–	Basidia 11–15 μm long	*** T. flammea ***
15	Basidia predominantly clavate	*** T. tawa ***
–	Basidia globose to subglobose or ellipsoid to oval	**16**
16	Conidia present	**17**
–	Conidia absent	**18**
17	Conidiogenous cells globose or subglobose to ellipsoid, basidiospore > 12 μm long	*** T. tropica ***
–	Conidiogenous cells phialide-like, basidiospore 6–9 μm long	*** T. armeniaca ***
18	Hollow lobes	*** T. erythrina ***
–	Not having hollow lobes	*** T. dysenterica ***

## Supplementary Material

XML Treatment for Tremella basidiomaticola

XML Treatment for Tremella cheejenii

XML Treatment for Tremella erythrina

XML Treatment for Tremella salmonea

## Supplementary materials

Supplementary material 1 Table S1. Physiological properties Data type: measurement This is an open access article distributed under the terms of the Creative Commons Attribution License (CC BY 4.0), which permits unrestricted use, distribution, and reproduction in any medium, provided the original author and source are credited.Ying Zhao, Xin-zhan Liu, Feng-yan Bai

Supplementary material 2 Sequence alignment Data type: phylogenetic data This is an open access article distributed under the terms of the Creative Commons Attribution License (CC BY 4.0), which permits unrestricted use, distribution, and reproduction in any medium, provided the original author and source are credited.Ying Zhao, Xin-zhan Liu, Feng-yan Bai
